# Mechanism exploration of Zoledronic acid combined with PD-1 in the treatment of hepatocellular carcinoma

**DOI:** 10.1007/s00262-024-03652-2

**Published:** 2024-03-02

**Authors:** Xinru Fan, Zijun Yan, Yunkai Lin, Qing Wang, Li Jiang, Xiaomeng Yao, Liwei Dong, Lei Chen, Tuan Zhao, Jieqiong Zhao, Heping Hu, Hui Wang

**Affiliations:** 1 Department of Hepatobiliary Medicine, Eastern Hepatobiliary Surgery Hospital, Second Military Medical University, Shanghai, 200438 China; 2 Faculty of Hepato-Biliary-Pancreatic Surgery, The First Medical Center of Chinese People’s Liberation, Army General Hospital, Beijing, 100039 China; 3 National Center for Liver Cancer, Shanghai, 201805 China; 4 Outpatient Department, Eastern Hepatobiliary Surgery Hospital, Second Military Medical University, Shanghai, 200438 China

**Keywords:** Hepatocellular carcinoma, Zoledronic acid, PD-1 antibody, Macrophage

## Abstract

How to increase the response of immune checkpoint inhibitors (ICIs) is a challenge. In clinical, we found that Zoledronic acid (ZA) may increase the anti-tumor effect of immunotherapy for hepatocellular carcinoma (HCC). To explore the underlying mechanism, we established a mouse model of HCC by subcutaneously injecting Hepa1-6 cell line. The result showed that the tumor volume in the ZA plus anti-PD-1 monocloning antibody (anti-PD-1 mAb) treatment groups was significantly smaller than that of control group, and the onset time of tumor inhibition was even shorter than that of the anti-PD-1 mAb group. Using flow cytometry (FC) to detect the proportion of major immune cell subsets in tumor tissues of each group of mice, we found that the synergistic anti-tumor effect of ZA and anti-PD-1 mAb may be related to ZA-induced polarization of macrophages toward the M1 phenotype. Next, we performed bulk RNA sequencing on tumor samples from different groups to obtain differentially expressed genes (DEGs), which were then input DEGs into pathway enrichment analysis. Data indicated that ZA participated in the M1-type polarization via ferroptosis-related pathways. Our results revealed how ZA involves in the anti-tumor effect of PD-1 monoclonal antibody and provided a potential therapeutic candidate for patients with HCC.

## Method

### A clinical case of combined application of ZA and anti-PD-1

The patient was a 60-year-old Chinese female diagnosed as HCC in Nov.2020, with Barcelona Clinic Liver Cancer (BCLC) stage A, China Liver Cancer Staging (CNLC) stage I a, and Child–Pugh score of 7, grade B. Although the patient presented with many symptoms including portal hypertension, ascites, and hypoproteinemia, she refused liver transplantation and underwent transarterial chemoembolization (TACE) treatments successively in the first two months and radioknife therapy in the sixth month, respectively. The bone metastasis from HCC appeared in the tenth month. She discontinued the Lenvatinib due to severe digestive symptoms and switched to immunotherapy. ZA (Zoledronic acid, Chia Tai Tianqing Pharmaceutical Group Co., Ltd.) was injected for the treatment of bone metastases at a dose of 4 minigramms, in synchrony with the injection of anti-PD-1 antibody (Tislelizumab Injection, Beigene Co., Ltd.)every 3–4 weeks. The sizes of the primary tumor and metastatic tumors were observed by magnetic resonance imaging (MRI). The clinically used tumor markers, such as alpha-fetoprotein (AFP), proteins induced by VK absence or antagonism (PIVKA), and carbohydrate antigen 19–9 (CA19-9) were detected. Circulating tumor cell (CTC) and CTC-micro embolism (CTM) were also detected as indicators of tumor metastases.

### Animal experiments

All animal experiments were performed following a reference protocol approved by the Ethics Board of the Eastern Hepatobiliary Surgery Hospital, Shanghai. BALB/c-nu/nu mice (6-week-old, female) were purchased from Shanghai Bikai Biotechnology Company (Shanghai, China). All mice were housed in a specific pathogen-free facility with a 12h light, 12h dark cycle and provide plenty of food and water. For cell line xenografts, Hepa1-6 cells (2 × 10^6^) in phosphate-buffered saline (PBS, Basalmedia) were subcutaneously injected into 6-week-old female nude mice. For ZA (Selleck,S1314) treatment, mice were given intravenous injections of vehicle (PBS, Basalmedia) or 100 μg/kg ZA every day or every other day. For anti-PD-1 mAb treatment, mice were given 1mg/kg body weight of anti-PD-1 mAb (Bio X Cell, BE0146) via intraperitoneal injection in 5th day after tumor cell inoculation, every two days. Controls were given vehicle (PBS, Basalmedia). Measure the body weight of mice in each group every 2 days. A caliper was used to measure tumor volume weekly, and tumor volume was calculated according to the formula: 0.5 × L × W^2^, where L is the longest diameter and W is the shortest diameter. In the end experiment, mice were sacrificed and tissue samples were collected.

### Murine cells isolation and flow cytometry

Tumor tissue was washed several times in PBS, finely chopped with a razor blade and digested in HBSS containing 1mg/ml Collagenase type IV (Sigma-Aldrich, C5138),100ug/ml DNase I (Sigma-Aldrich, D5319), and 5% fetal calf serum for 10min at 37 ℃ with gentle rotation in shaker (150 rpm). Leukocytes were isolated from the supernatant with Percoll (Solarbio, P8370) gradient separation method in which the cells were responded in 40% Percoll and underlayered with 80% Percoll followed by centrifugation at 2500 rpm for 20min. For surface marker staining, cells were washed with PBS containing 0.5% BSA and stained with CD45-BV605 (103,140, Biolegend), F4/80-APC-cy7 (123,117, Biolegend), CD11b-APC (17-0112-82, eBioscience), CD11c-PE (12-0114-81, eBioscience), CD206-BV421 (141,717, Biolegend), Ly6G-PE-cy7 (127,617, Biolegend), CD3e-PerCP (100,325, Biolegend), CD4-AF700 (100,430, Biolegend) and CD8-FITC (11-0081-82, eBioscience) for 30 min in the dark. Serum levels of Data acquisition were performed on an LSR Fortessa instrument (BD Biosciences) and analyzed by using FlowJo software (Treestar) and SPSS26.0. IL-18 and IFN-γ were detected by enzyme-linked immunosorbent assay (ELISA, MultiScience).

### Histological analysis

For immunohistochemistry, the tissues (liver, kidney, lung, and tumor) were fixed overnight in 10% formalin, embedded in paraffin and cut into 5 μm sections. The tumor samples were stained with Ki67 (ab21700, Abcam). After overnight incubation, the slides were washed and incubated with the secondary antibody (HRP-Polymer, Biocare Medical) for 30 min at room temperature. The slides were washed three times and stained with 3, 3’-diaminobenzidine (DAB) substrate (Thermo Fisher Scientific). The slides then were counterstained with hematoxylin and mounted with a mounting medium. In addition, the hematoxylin–eosin (HE) staining was performed on paraffin-embedded tissue sections by the ST5010 Autostainer XL (Leica). Images were obtained by the Aperio Image Scope Viewer (Leica). Quantification by counting of Ki67^+^ cells number in random area per sample.

### Data collection of HCC cohorts

Three groups of bulk RNA sequencing data from HCC patients were included: TCGA-LIHC (*n* = 373), ICGC-LICA-JP (*n* = 231), and ICGC-LICA-FR (*n* = 161). Normalized datasets generated by Illumina were downloaded from the Cancer Genome Atlas (TCGA, https://portal.gdc.cancer.gov/) and International Cancer Genome Consortium (ICGC, https://dcc.icgc.org/). Only tumor samples were incorporated into the study.

### Evaluation of immune infiltration in HCC cohorts

The ESTIMATE [1] algorithm was applied to calculate the degree of immune infiltration in the tumor microenvironment. ESTIMATE scores and tumor purity were presented in the study. Based on the calculation result, we identified the median of ESTIMATE scores and tumor purity as optimal cutoff and used it to categorize patient data into the high and low immunogenicity group for further analysis.

### Prediction of the drug sensitivity in HCC cohorts

The oncoPredict [2] package was utilized to predict the half-maximal inhibitory concentration (IC50) of sorafenib and Zoledronic acid in HCC patients. The source of drug sensitivity databases is Genomics of Drug Sensitivity in Cancer (GDSC) [3]. GDSCv2 builds upon GDSCv1 by incorporating additional cell lines and a broader range of drugs, so we used GDSCv2 here. oncoPredict collects tumor cell sensitivity and response to drugs, thus uses gene expression data of tumor cell lines as the training set [2]. The GDSCv2 gene expression profile and corresponding drug response information were downloaded to calculate IC50s of 196 compounds or drug monomers by calculating the bulk RNA sequencing data. When executing the calcPhenotype function in the oncoPredict package, the batchCorrect method was designated as ‘eb’, and any genes exhibiting less than 20 percent viability were excluded. All other parameters were retained at their default settings.

### Statistical analysis

The bioinformation statistical analyses were conducted via R (https://www.r-project.org/). Boxplots were constructed to visualize differences via graphpad8.0. Student’s *t* test was utilized for the comparison between two groups, while one-way analysis of variance (ANOVA) was employed for analyzing the differences among three groups. The flow cytometry data was analyzed by using FlowJo software (Treestar) and SPSS26.0. *P* values were labeled as *, *p* < 0.05; **, *p* < 0.01; ***, *p* < 0.001; and ****, *p* < 0.0001. A *p* value below 0.05 was deemed statistically significant.

## Backgrounds

Surgery is the preferred treatment for HCC, with a 5-year survival rate of about 70–80%, but only a small proportion of patients are suitable for surgery treatment [4]. For unresectable HCC, TACE was the most common treatment until 20 years ago [5]. The emergence of immune checkpoint blockade (ICB) has upended the traditional notion of tumor therapy. Immunotherapy does not directly attack tumor cells, but activate the immune system [6]. However, the response rate of immunotherapy is still a problem, and the efficacy is limited by the tumor immune microenvironment (TIME). Recently, the combination of immune checkpoint inhibitors with common drugs was applied to improve the response rate.

Bisphosphonates (BP) are initially used to treat osteoporosis [7], since they lead to inhibition of osteoclast function and ultimately induction of osteoclast apoptosis. With in-depth research on BPs, their anti-tumor properties have been gradually explored, including inhibiting tumor cell adhesion, migration and angiogenesis [8]. Zoledronic acid (ZA, C5H10N2O7P2), also called zoledronate, is the third generation of BP with a history of only 25 years, belonging to nitrogen-containing bisphosphonate (N-BP). ZA has been currently employed as a  BP to treat osteoporosis and reduce the risk of bone fractures [9]. ZA forms tertiary amines on the side chain of R2, which has a stronger inhibitory effect on farnesyl pyrophosphate synthase (FPPS) and shows stronger inhibition of bone resorption [10]. ZA has been clinically proven to be applicable to treat multiple myeloma and bone metastases of various cancers including breast, prostate, lung cancers [11–13]. In addition, ZA has become an adjuvant therapy for early breast cancer [14, 15]. As an anti-resorptive drug, ZA has been found to directly inhibit tumors and improve immunosurveillance against tumor [16–18], However, the underlying molecular mechanisms remain elusive. Previous research suggested that ZA induces the colonization of gamma delta T-cells in HCC [19] and inhibited the polarization of tumor-associated macrophages (TAMs) from M1 to M2 phenotype [20, 21]. Therefore, ZA may potently modulate the tumor immune microenvironment to increase the immunotherapeutic efficacy. A study showed that the blockade of PD-1 in combination with ZA enhanced the anti-tumor efficacy in a mouse model of breast cancer [22]. However, the therapeutic effects of ZA on liver cancer have not been explored. Here, we report a case of tislelizumab combined with ZA in the treatment of HCC with bone metastasis. In this study, we found that the combination of PD-1 antibody and ZA has a synergistic anti-tumor effect in vitro. ZA may increase the anti-tumor effect of immunotherapy for HCC.

## Result

### A case of combined use of ZA and anti-PD-1 in the treatment of HCC

**Fig. 1:**
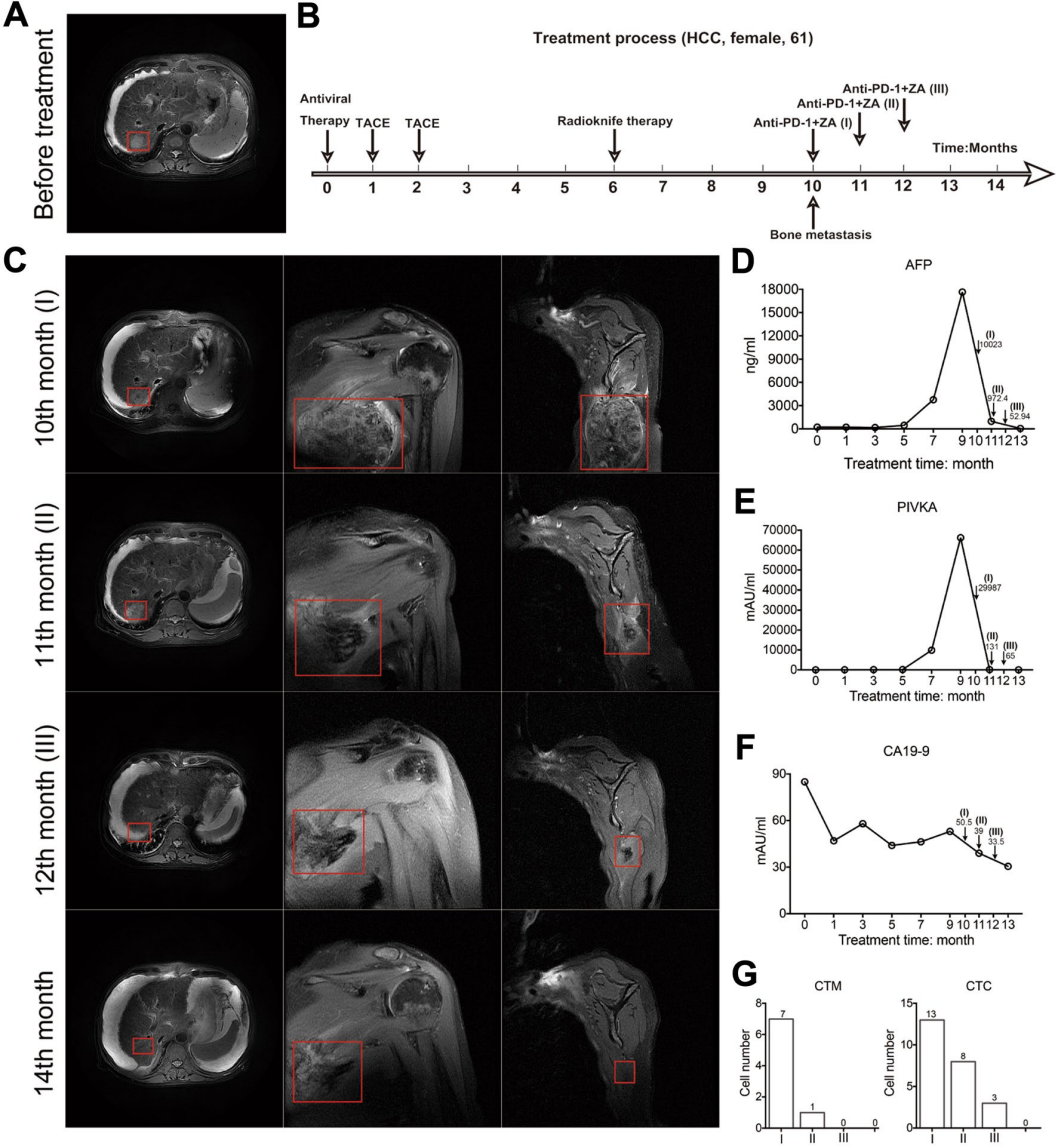
The case of combined ZA and anti-PD-1 in the treatment of bone metastasis from liver cancer. **A** MRI of the primary lesion of the liver cancer before treatment. **B** The process of treatment. (Antiviral therapy was given within the first month after the diagnosis of primary liver cancer.) The patient received two TACE treatments in the first and second month. At the 6th month, radioknife treatment was used for the patient. Bone metastasis was found at the 10th month. The anti-PD-1 treatments combined with ZA were applied in the 10th, 11th and 12th months, respectively. **C** MRI images of the liver and left scapula after the patient was found to have bone metastasis. At the 11th month, the left scapular lesion was significantly reduced on the 7th day after treatment. At the 12th month, the lesion in the right lobe of the liver was stable, and the metastasis in the left scapula was significantly reduced. At the 14th month, there was no progression of the intrahepatic lesion, and the extrahepatic metastasis almost disappeared. **D**, **E** High AFP and PIVKA were detected and subsequently reaching maximum values when bone metastasis was observed. Both of them decreased gradually after anti-PD-1 combined ZA treatment. **F** The patient had a high level of CA19-9 at diagnosis. From the beginning to the tenth month, the level of CA19-9 gradually decreased but remained above normal. And the rate of decrease gradually slowed down or even slightly increased. After the third administration of anti-PD-1 + ZA, CA19-9 decreased to the normal range (< 37mAU/ml). **G** With the application of the combined treatment, CTC and CTM were gradually reduced to 0

A 60-year-old female was diagnosed as HCC with complications including portal hypertension, ascites, and hypoproteinemia. The liver MRI demonstrated a low-signal nodule on T1-weighted imaging (T1WI) and a high-signal nodule on T2-weighted imaging (T2WI) in the right liver lobe (Fig. 1A). The contrast enhanced MRI showed significantly intensive signal in the arterial phase and the size of the nodule was about 1.8cm × 2.4cm. As the patient refused liver transplantation, she underwent two TACE treatments in the first two months and radioknife therapy in the sixth month after anti-hepatitis C virus (HCV) viral therapy (Fig. 1B). Due to the MRI of the patient’s shoulder and upper arm joint showing metastatic lesion in the left scapula in the 10th month, we started the combination therapy of PD-1 antibody and ZA in the tenth month. Surprisingly, shrinkage of extrahepatic metastasis was observed only one week after the first combined application. The size of tumor metastasis was gradually reduced during the progression (Fig. 1C).

The patient was also found to have high AFP and PIVKA at the time of diagnosis of primary liver cancer (Fig. 1D, E). Both indicators decreased gradually after combination therapy of PD-1 antibody and ZA. The concentration of CA19-9 fluctuated downward and eventually fell to the range of normal values (Fig. 1G). Furthermore, 13 CTCs and 7 clusters/4 ml of CTM were detected at the time of bone metastasis (Fig. 1G). The value of CTCs and CTM gradually decreased to zero over the course of three treatments. The case enlightened us that the combination of PD-1 antibodies and ZA for the treatment of HCC may have a synergistic effect in inhibiting tumor growth, at least for some patients.

### ZA shows sensitivity to patients with HCC from TCGA and ICGC datasets

**Fig. 2:**
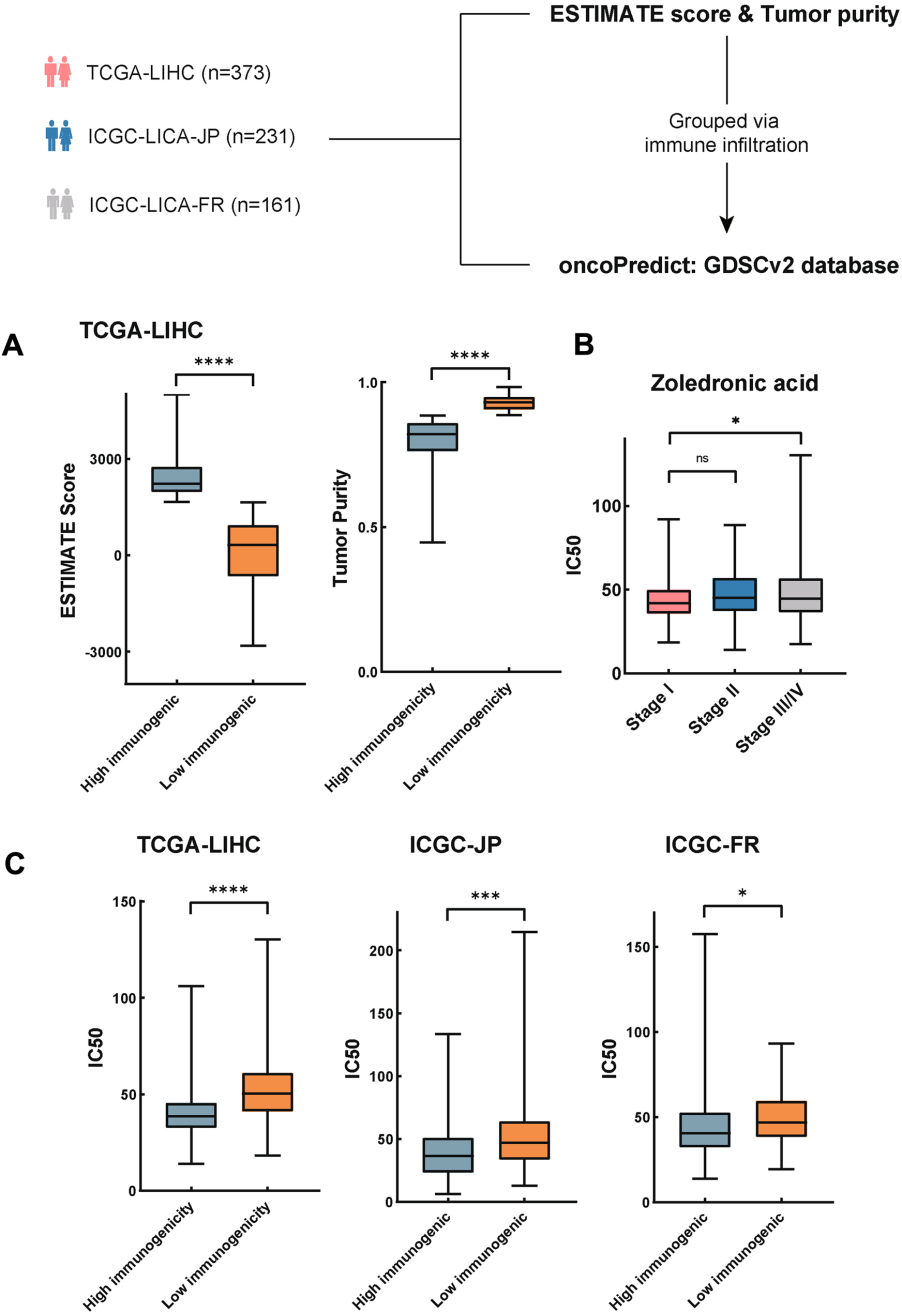
Evaluating the effect of tumor immune infiltration and tumor purity on the drug sensitivity of ZA based on patients with HCC in TCGA and ICGC datasets. **A** The flowchart of data research. TCGA-LIHC (*n* = 373), ICGC-LICA-JP (*n* = 231), and ICGC-LICA-FR (*n* = 161) were included into analyses. The ESTIMATE score and tumor purity were first calculated. **B** Boxplots showed the patient with HCC who had higher degree of immune infiltration had significantly higher ESTIMATE score and lower tumor purity in TCGA-LIHC cohort(****, *p* < 0.0001). **C** The boxplot showed prediction of the drug sensitivity in TCGA-LIHC cohort. The value of IC50 were lower in stage I and stage II than stage III (*, *p* < 0.05). **D** Boxplots showed the values of IC50 were lower in HCC patients with high immunogenicity (TCGA-LIHC ****, *p* < 0.0001; ICGC-JP ***, *p* < 0.001; ICGC-FR *p < 0.05)

The drug sensitivity of HCC patients to ZA was calculated via oncoPredict [2] in silico. We first collected three HCC cohorts, including TCGA-LIHC, ICGC-JP, and ICGC-FR (Fig. 2A). Tumor samples with bulk RNA sequencing processed data were downloaded and analyzed. Based on the GDSCv2 drug prediction database [2], the IC50s of total 765 tumor samples from three cohorts against 198 small molecule compounds or drug monomers were evaluated. Sorafenib, the first-line drug for the treatment of HCC, was enumerated to compare with ZA (Table 1). The value of IC50 and ranking of sorafenib and ZA in three cohorts were close to each other. Although ZA has a higher ranking and the IC50 value than sorafenib, ZA still possesses the potential to treat HCC based on the stable and relatively low IC50 and ranking. Then, the immune infiltration of incorporated samples was evaluated by the ESTIMATE score and tumor purity [1]. The medians were set as cut-off value to divide the samples into high- and low-immunogenic group in three cohorts, respectively. The ESTIMATE score and tumor purity of TCGA-LIHC were exhibited in Fig. 2B. Interestingly, the values of IC50 in the high-immunogenic group were lower than that in low-immunogenic group (Fig. 2D), indicating better drug efficacy in the former one. In addition, we grouped samples from TCGA-LIHC via AJCC TNM stage. The values of IC50 were significantly lower in stage I (Fig. 2C). The result indicated that administration of ZA in the early stage of HCC acquired better efficacy than the late stage. In conclusion, ZA exerted better anti-tumor efficacy in the tumor immune microenvironment with higher degree of immune infiltration.

**Table 1:** IC50 of Sorafenib and Zoledronate in TCGA-LIHC, ICGC-JP, and ICGC-FR

	TCGA-LIHC	ICGC-JP	ICGC-FR
*Sorafenib*			
Average	15.22	20.86	15.50
Median	14.38	16.41	13.54
Stdeva	5.33	20.27	7.87
Rank	80/198	59/198	67/198
*Zoledronate*			
Average	46.83	48.84	46.36
Median	43.69	40.44	43.91
Stdeva	15.72	32.98	18.46
Rank	129/198	98/198	116/198

### The combination of ZA and ICI possesses synergistic anti-tumor efficacy in vitro

**Fig. 3:**
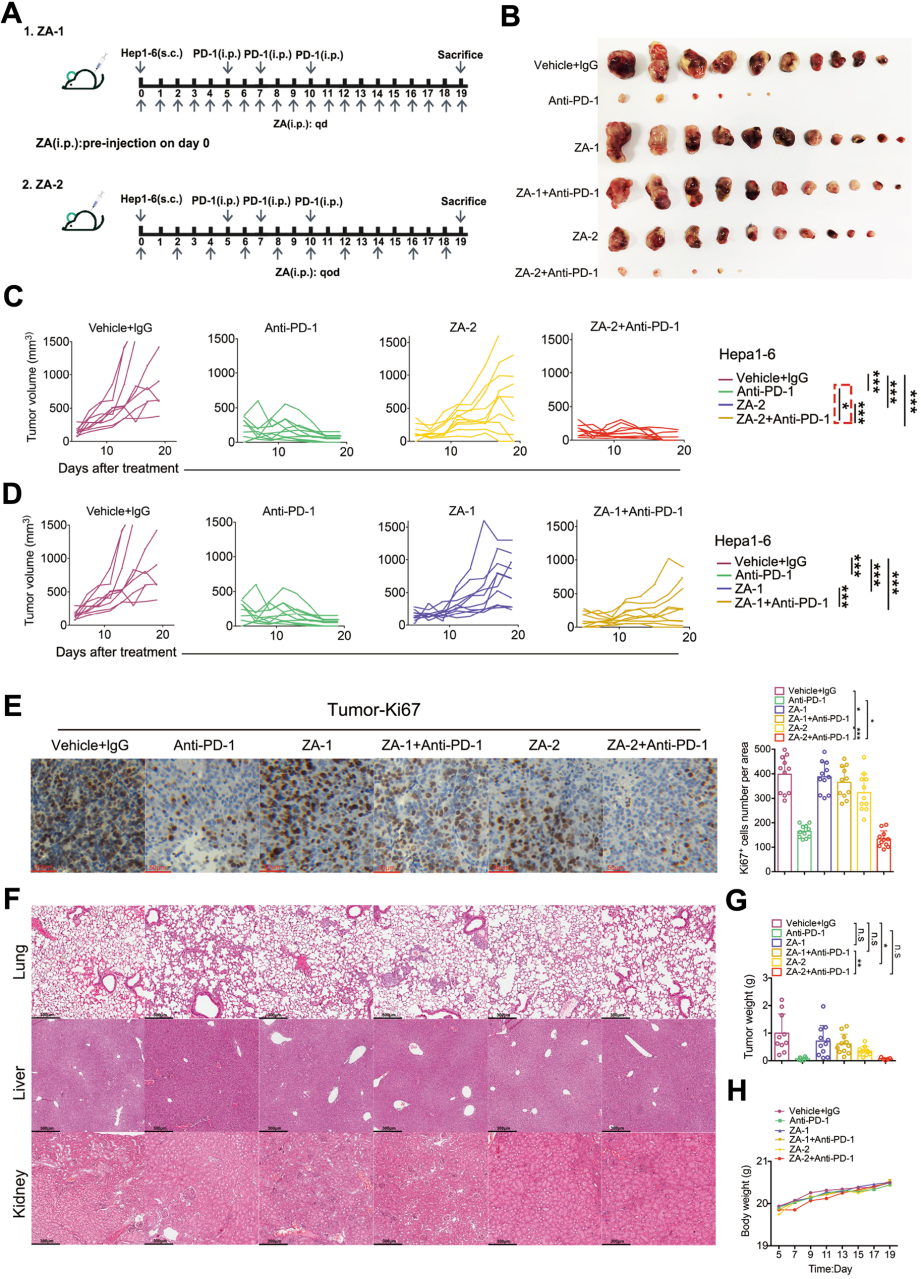
The combination of ZA and ICI has synergistic anti-tumor efficacy in vitro. **A** Schematic diagram of HCC modeling in mice. Mice were defined as ZA-1 and ZA-2 based on different administration frequencies. **B** The animal experiments were grouped into combined application of ZA and anti-PD-1, only use anti-PD-1 or ZA, and control group. More than ten mice were included into each group. **C**, **D** Tumor volumes of mice in each group were monitored (labeled as *, *p* < 0.05; ***, *p* < 0.001). **E** Representative immunohistochemical images of tumor tissues stained with Ki67 from each group. The quantification of Ki67 + cells number per area was counted and calculated (labeled as *, *p* < 0.05; ***, *p* < 0.001). **F** Representative HE staining images of lung, liver, and kidney tissues were taken from each group. **G**, **H** Tumor weight and body weight were recorded and compared between each group during the progression of tumor (labeled as *, *p* < 0.05; **, *p* < 0.01)

To evaluate the synergistic anti-tumor effect of ZA combined with anti-PD-1 mAb, we established a mouse model of HCC by subcutaneously injecting Hepa1-6 cell line (Fig. 3A). ZA was intraperitoneal injected every day (referred to as ZA-1) or every other day (referred to as ZA-2). The tumor volume of mice in each group was measured daily during the observation period of 20 days. Mice were euthanized on the 20th days, and tumor masses were collected (Fig. 3B). The tumor volume in the control group increased significantly over time, and the tumor volume in the ZA-2 single treatment group increased relatively slowly compared with the control group (*P* < 0.001) (Fig. 3B, C), suggesting the anti-tumor effect of ZA. The tumor volumes of the anti-PD-1 mAb treatment group (referred to as anti-PD-1) and ZA-2 plus anti-PD-1 mAb treatment groups (referred to as ZA-2 + anti-PD-1) were significantly smaller than those of the control group (*P* < 0.001) (Fig. 3B). There was no significant difference in tumor weight between the two groups (*P* = 0.32) (Fig. 3G). However, the ZA-2 + anti-PD-1 group had a shorter onset time for tumor inhibition(*P* < 0.05) (Fig. 3C). These results suggested that ZA and anti-PD-1 mAb have a synergistic anti-tumor effect. Although increasing the frequency of ZA administration resulted in relatively slow growth of tumor volume in the ZA-1 single treatment group compared with the control group (*P* < 0.001). However, tumor volume in the ZA-1 plus anti-PD-1 mAb treatment groups (referred to as ZA-1 + anti-PD-1) was larger than that in the anti-PD-1 group (*P* < 0.01) (Fig. 3D). Moreover, after 20 days of observation, the tumor weight in the ZA-1 + anti-PD-1 group was larger than that in the ZA-2 + anti-PD-1 group (*P* < 0.05) (Fig. 3G). The above results suggested that increasing the frequency of ZA administration actually attenuated the potential synergistic anti-tumor effect between ZA and anti-PD-1 antibody. Next, we detected the proliferation marker Ki67 of tumor tissue in each group. Consistent with the difference in tumor growth, the expression level of Ki67 was significantly reduced in ZA-2 + anti-PD-1 group than anti-PD-1 group (Fig. 3E). To evaluate the safety of the treatment, we examined the morphology main organs and body weight of mice during the 20-day observation period. The results indicated that no significant lesions or metastatic tumor foci were observed in liver, kidney, and lung tissue sections (Fig. 3F). In addition, no significant weight loss of mice in each group (Fig. 3H).

### ZA participates in the M1-type polarization of macrophages in TIME

**Fig. 4:**
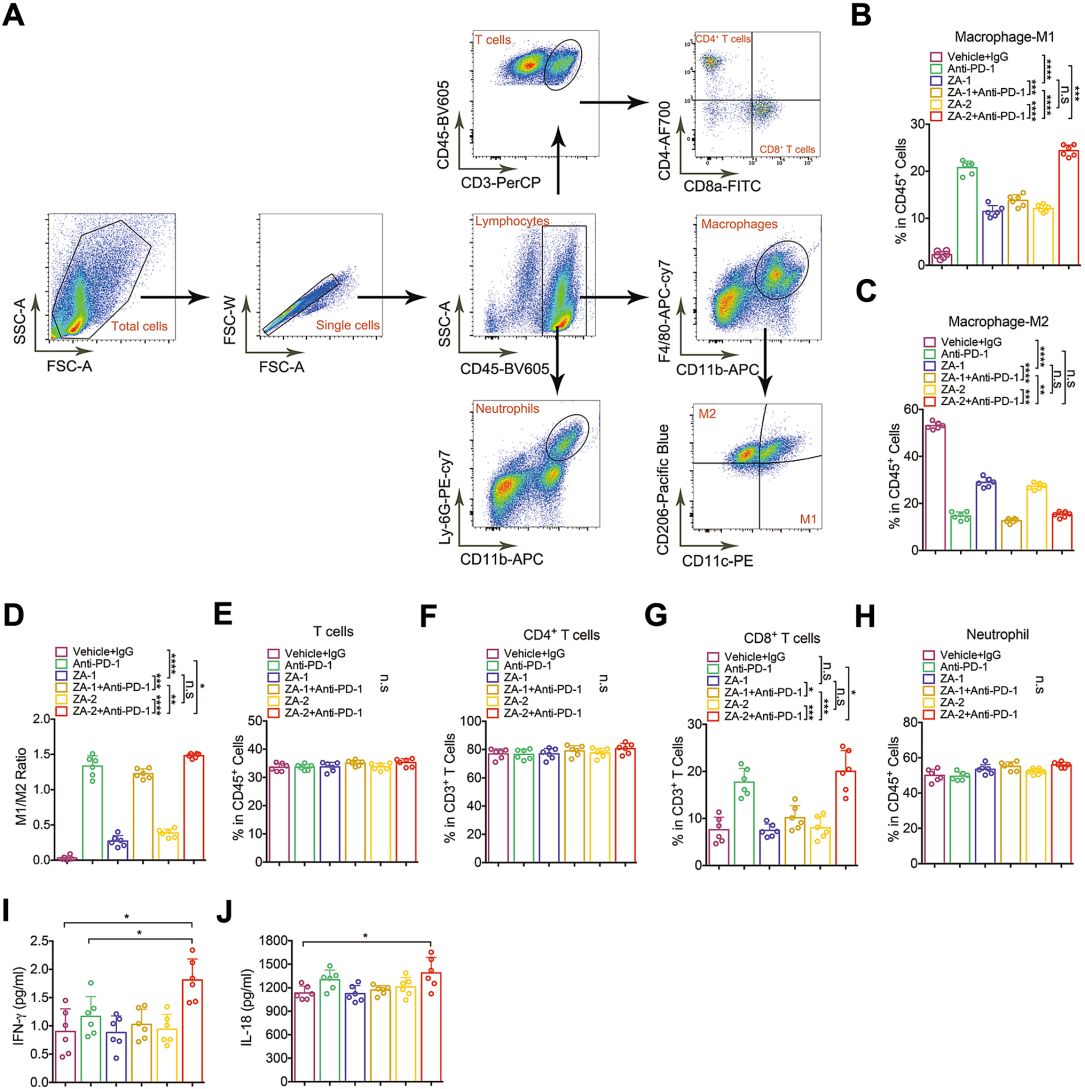
Changes in the proportion of immune cells analyzed by flow cytometry. **A** Representative flow cytometric images showed gating strategy and immune cell clustering. **B** Bar chart showed the proportion of M1 macrophages in CD45 + cells of each group. (labeled as ***, *p* < 0.001; ****, *p* < 0.0001). **C** Bar chart showed the cell proportion of M2 macrophages in CD45 + cells of each group(labeled as **, *p* < 0.01; ***, *p* < 0.001; ****, *p* < 0.0001). **D** Bar chart showed M1/M2 ratio in each group(labeled as *, *p* < 0.05; **, *p* < 0.01; ***, *p* < 0.001; ****, *p* < 0.0001). **E**–**H** Bar chart showed the cell proportion of T cells, CD4 + T cells, CD8 + T cells, and neutrophils in CD45 + cells (labeled as *, *p* < 0.05; ***, *p* < 0.001). **I**, **J** Bar chart showed the plasma concentration of IL-18 and IFN-*γ* in each group (*, *p* < 0.05)

The immune cells were sorted by FC and stained with markers of myeloid and lymphoid cells (Fig. 4A). Myeloid and lymphoid linages were compared in tumor samples of each group. M1 macrophages were noted as CD11b + F4/80 + /CD11C + , and M2 macrophages were noted as CD11b + /F4/80 + /CD206 + . Compared with the ZA groups, the expression of M1 macrophage marker CD11C remarkably increased in the ZA + anti-PD-1 groups (Fig. 4B). In addition, we found a significant difference in the proportion of M1 macrophage between with ZA-1 + anti-PD-1 and ZA-2 + anti-PD-1 group (Fig. 4B). These results suggested that ZA may be involved in macrophage polarization, which was also influenced by the frequency of administration. A similar analysis of M2 macrophages confirmed this observation (Fig. 4C). Notably, the proportion of M1 macrophages in the ZA-2 + anti-PD-1 group was significantly higher than that in the anti-PD-1 group (Fig. 4B), while the percentage of M2 macrophages was similar in above two groups (Fig. 4C), indicating that ZA may promote M1-type macrophage polarization but not M2-type macrophage polarization. Consistent with this, the M1/M2 ratio was increased in ZA-2 + anti-PD-1 group compared to anti-PD-1 group (Fig. 4D). Moreover, we failed to find any changes in the proportion of T cells (CD45 + CD3 +) (Fig. 4E), CD4 + T cells (CD45 + CD3 + CD4) (Fig. 4F) and neutrophils (CD45/CD11b/Ly6G +) (Fig. 4H). Studies have shown that the synergistic anti-tumor effect of ZA and anti-PD-1 monoclonal antibody may be related to the enhanced CD8 + cells infiltration into tumors. We similarly observed the increased percentage of CD8 + T cells in the CD3 + T cell population in the ZA-2 + anti-PD-1 group compared to the anti-PD-1 groups. To further verify the effect of increased infiltration of CD8 + T cells after ZA plus anti-PD-1 mAb treatment, we examined the IFN-*γ* and IL-18 levels in the plasma of each group. There was significant increase in IFN-*γ *concentration in the ZA-2 + anti-PD-1 group when compared to either the control group or anti-PD-1 group (Fig. 4I). The result of IL-18 showed that there was significant increase only between ZA-2 + anti-PD-1 group and the control group (Fig. 4J). These findings suggested that ZA promote synergistic anti-tumor effects by enhancing M1 polarization and CD8^+^ cells infiltration.

### Transformations of macrophages may be related to ferroptosis-related signaling pathways

**Fig. 5:**
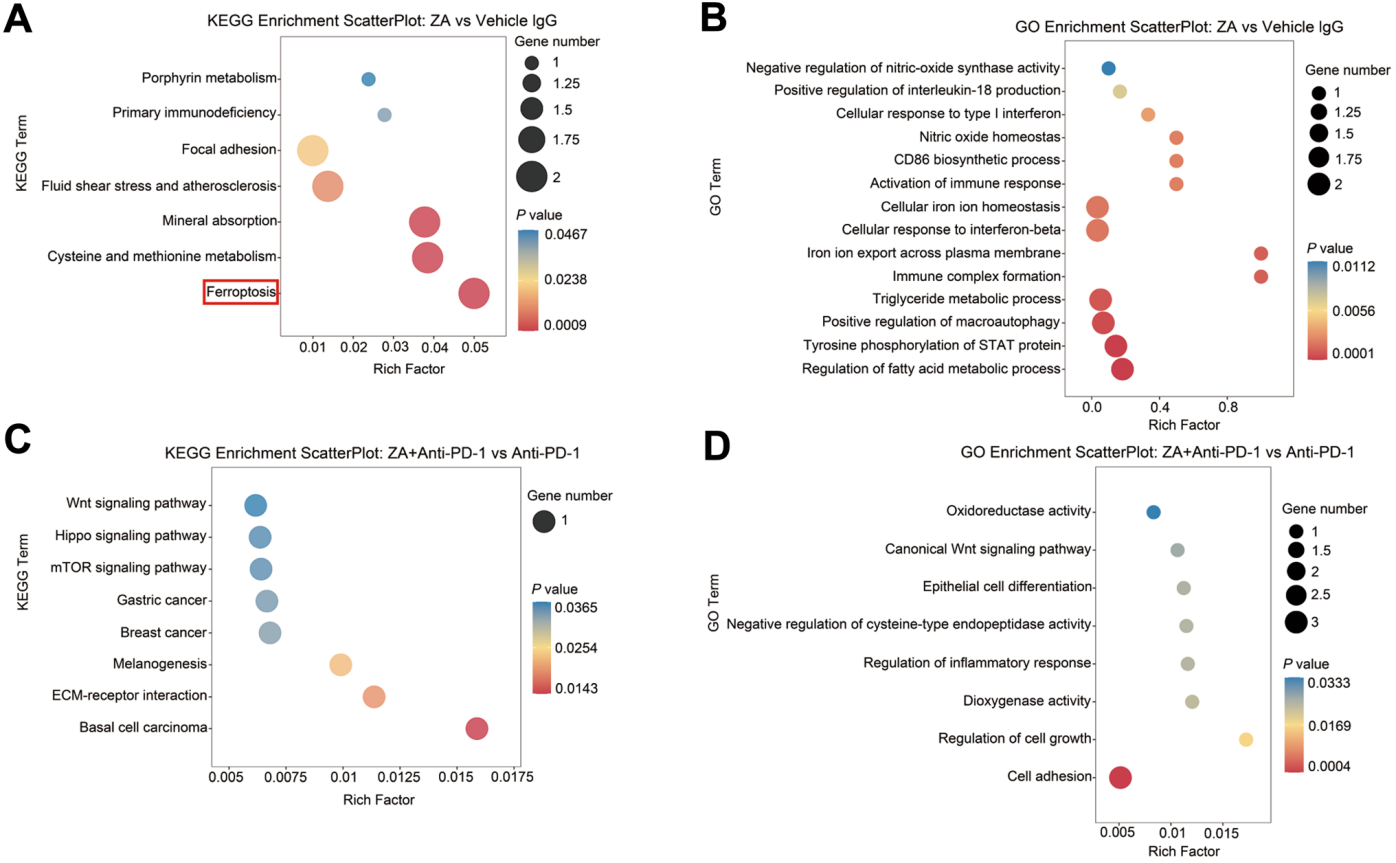
Ferroptosis-related pathway leading to the conversion of macrophages Bubble plots of KEGG and GO enrichment analyses. **A**, **B** DEGs between the application of ZA group and control group were input into KEGG and GO pathway datasets. Bubble plots showed DEGs were enriched in ferroptosis-related pathway. **C**, **D** DEGs between anti-PD-1 group and the combination of ZA and anti-PD-1 group were input into KEGG and GO pathway datasets

We performed bulk RNA sequencing on different groups of samples to obtain DEGs and input DEGs into pathway enrichment analysis. Compared with the control group, DEGs were enriched in ferroptosis-related pathway in the group treated with ZA. DEGs were enriched in Ferroptosis pathway in KEGG pathway dataset (Fig. 5A) and metabolism related pathway in GO datasets (Fig. 5B). Then, ZA + Anti-PD-1 group was compared with the group using single Anti-PD-1. KEGG enrichment analysis showed that Melanogenesis, ECM-receptor interaction and Basal cell carcinoma were the highest enriched pathway with significant P values (Fig. 5C), while cell adhesion was the most highly enriched pathway in GO enrichment analysis (Fig. 5D). These results suggested ZA participated in the M1-type polarization of via ferroptosis-related pathways.

## Discussion

BPs were first synthesized in the late nineteenth century. Initially used for industrial production, its clinical value was not discovered until the late 1960s [8]. The hydroxyl group in the R1 side chain of can be chelated with a calcium ion bidentate or tridentate coordination, giving them a high affinity for hydroxyapatite. Hydroxyapatite is the main inorganic component of human bone, and  BPs can selectively and rapidly bind to bone hydroxyapatite in vivo [23]. BPs are internalized during bone resorption through the endocytic activity of osteoclasts. By inhibiting FPPS in the mevalonate pathway, BPs inhibit the activity of osteoclasts and inhibit bone resorption [7].

In recent years, the anti-tumor properties of BPs have been gradually discovered. The second generation of BPs is able to inhibit the FPPS in the mevalonate pathway, thereby inhibiting the synthesis of farnesyl pyrophosphate (FPP) and geranyl pyrophosphate (GGPP) downstream. However, FPP and GGPP are involved in the key step of protein isoprenylation, which is a necessary step for GTPase modification. GTPase is a crucial intracellular signaling protein, which plays a key role in cell migration, movement and adhesion [10]. In addition, inhibition of FPPS leads to the accumulation of upstream isopentenyl pyrophosphate (IPP). This leads to the formation of an ATP analogue, which is able to induce apoptosis by blocking mitochondrial ADP/ATP translocase [24]. As a third-generation BP, ZA has a stronger effect as mentioned above. 

The incidence of bone metastasis (BM) from HCC is increasing gradually with prolonged survival period due to the improvement of diagnostic techniques and treatment methods [25]. A previous study noted that BM from HCC accounted for about 30% of extrahepatic metastases [26]. Studies on the BM in HCC are relatively few compared with those on BM in breast, prostate, and lung cancers. Owing to this limitation, optimal treatment strategies are not well defined. ZA is clinically used to treat bone metastases combined system treatment. In this case report, we observed that the obvious shrinkage of the metastatic tumor volume on the left scapula only one week after the combination treatment of PD-1 antibody and ZA. This treatment response time was much shorter than the average treatment response time of PD-1 antibody. We consider that the efficacy of bone metastases in this case is not only related to ZA as a novel anti-absorption BP drug, but also to ZA anti-tumor mechanism, especially combination with PD-1. As introduced in the background, ZA is stable pyrophosphate analogues, where a carbon atom replaces the central oxygen atom, making the P-C-P backbone non-hydrolysable. Furthermore, the P-C-P backbone structure allows the BP binding to hydroxyapatite in bone tissue through the chelation of Ca2 + . Hydroxyapatite is the main inorganic component of human bone, and BPs can selectively and rapidly bind to bone hydroxyapatite in vivo [23]. This is why ZA is typically used for bone metastases in patients but not for other metastatic or localized stages. It is found that ZA has direct anti-tumor efficacy and is often treated as a combination of Early Breast Cancer. ZA can inhibit the differentiation and apoptosis of osteoclast, suppress metastasis of breast cancer and inhibit angiogenesis [23]. At present, there is no report clinically about combination immunotherapy of ZA in the HCC treatment. A study on ZA immunotherapy in a mouse model of breast cancer suggested that ZA combined with PD-1 treatment could improve immune efficacy by recovering CD8 + cell number [22]. Currently, fundamental researches [19, 21, 27] on ZA improving the immune microenvironment focused on modulating various immune cells including Treg cells, tissue-resident memory T-cells (TRM) and TAMs, besides CD8 + cells. In this clinical case, we found that the HCC patient with bone metastasis showed partial remission (PR) after using the combination of ZA and PD-1. We explored potent related mechanisms by building a mouse model of HCC, the results suggested that it may be related to enhancing M1 polarization of TAMs and CD8 + cells infiltration. We are expecting to provide the possibility for the new combination immunotherapy with ZA for HCC with bone metastasis. The specific mechanism still needs deeply exploration and evidence.

In this experiment study, PD-1 blockade alone or ZA alone significantly slowed tumor growth, showing promising anti-tumor effects. In addition, we found that PD-1 monoclonal antibody combined with ZA had the earliest onset time compared with other groups (*P* < 0.05). There may be a synergistic anti-tumor mechanism between ZA and anti-PD-1. Studies related to the treatment of HCC with anti-PD-1 monoclonal antibodies in combination with ZA have not been reported. Our study suggests that the mechanism may be related to the polarization of macrophages to M1-type induced by ZA. Changes in the microenvironment may cause macrophages to switch between the two phenotypes or become a hybrid of the two cells [28]. M1 macrophages have proinflammatory effect and strong antigen presenting ability. Polarization was induced by Interferon-*γ* (IFN-*γ*), lipopolysaccharide (LPS) and other factors. It can secrete a large number of proinflammatory cytokines such as interleukin (IL)-1*β*, Inducible nitric oxide synthase (iNOS), tumor necrosis factor-a (TNF-a), IL-12, to promote T helper (Th)1 response [29]. M1 macrophages inhibit cell proliferation and cause tissue damage and may participate in anti-tumor immune response [30]. In our study, the expression of M1-type macrophage marker CD11C in all groups significantly increased compared with the control group, suggesting that M1-type macrophages have anti-tumor effects. On the other hand, M2 macrophages have anti-inflammatory effects and poor ability to present antigens, which play a key role in the dynamic balance of immune function. It promotes Th2 response, wound healing and tissue regeneration [28]. Therefore, M2 macrophages play an important role in tumor growth. Tumor tissue recruits circulating macrophages to the TME and polarizes them to the M2 phenotype to become TAMs. TAMs promote tumor progression by promoting angiogenesis, immunosuppression, and metastasis [30]. In this study, the expression of the M2-type macrophage marker CD206 was significantly higher in the control group than those in the rest of the groups. It is suggested that M2-type macrophages can promote tumor. Fortunately, TAMs are highly malleable. Phenotypic remodeling of TAMs may effectively alleviate tumor immunosuppression to achieve adequate tumor immunotherapy. We found that compared with anti-PD-1 group, the expression of CD11C in ZA-2 + anti-PD-1 group was increased (*P* < 0.001). These results suggested that the synergistic anti-tumor effect of ZA and anti-PD-1 may be related to the polarization of macrophages to M1 phenotype induced by ZA.

The effectiveness of immunotherapy depends not only on the choice of medication, but also on the dosage, interval and method of administration. There is currently no consensus on whether the dosage and interval time of administration can be changed. In this paper, we surprisingly found that the interval of administration can affect the outcome of combination therapy, as evidenced by the obvious contrast in tumor growth between the “every day” group and “every other day” group of ZA administration. For solid tumors, the therapeutic effect is related to the plasma concentrations, the tumor penetration rate of drugs and its distribution within tumors. However, compared to the “every other day” administration group, the higher drug plasma concentrations in the “every day” administration group actually attenuated the potential synergistic anti-tumor effect. This indicated that the influencing factors of the anti-tumor effect of immunotherapy need to be further explored. Cancer immunotherapy is the process of restarting and maintaining the tumor immune cycle to restore normal anti-tumor immune responses. We believe that the frequency of administration may regulate the anti-tumor immune responses by modifying the immune microenvironment within tumor, thereby affecting the final treatment. It may involve the M1/M2 polarization, T cell differentiation and neutrophil activation, which requires follow-up verification on animal models and humans.

ZA may cause a rare but serious complication noted as bisphosphonate-related osteonecrosis of the jaw (BRONJ). BRONJ was defined as exposed necrotic bone in the maxillofacial region lasting more than 8 weeks, without history of radiation treatment to the jaw or treatment with an antiresorptive or antiangiogenic agent [31]. The specific mechanism of BRONJ is still unclear, and it may be related to the polarization of macrophages [32–34]. Qunzhou Zhang et al. found that Th17 cells, IL-17 cytokines and M1-type macrophages increased at BRONJ injury sites in mice and humans. By blocking IL-17 activity, M1 macrophages decreased significantly. This study proposed that ZA might induce M1-type polarization of macrophages by promoting IL-17 secretion, thereby promoting INF-*γ*-mediated signal transducer and activator of transcription (STAT)-1 signaling pathway [33]. Weiwen Zhu et al. found that ZA-induced polarization of M1 macrophages was accompanied by an increase in toll-like receptor 4 (TLR)-4 expression. TLR-4 is a key receptor that regulates the innate immune system. This study suggested that ZA might induce the polarization of M1 macrophages by activating TLR-4 and its downstream nuclear factor kappa B (NF-kB) signaling pathway [34]. In summary, although the specific mechanism is not clear, it can be determined that ZA can induce macrophages to M1-type polarization. This is consistent with our findings.

ZA reaches peak concentration Cmax at the end of intravenous administration. It quickly drops to 10% of Cmax after 4h and 1% of Cmax after 24h [7]. 39% of the dose (in cancer patients) is excreted from the kidneys within 24 h, with the remainder being bound mainly to bone [7]. Hortobagyi GN et al. found that in patients with bone metastases from breast cancer, for the prevention of bone-related events (SRE), the use of ZA every 3 months was no less effective than once a month [35]. However, there are no definitive studies on the optimal dose and frequency of ZA administration in patients with bone metastases from HCC. ZA is usually clinically administered every 3 or 4 weeks. To investigate the effect of different dosing frequencies on the anti-tumor effect of ZA, we increased the dosing frequency of ZA from every other day to daily dosing. Notably, increasing the frequency of ZA dosing appeared to diminish the potential synergistic anti-tumor effects between ZA and anti-PD-1 monoclonal antibodies. The exact mechanism is unclear and deserves further exploration.

In conclusion, our research shows that the combination of anti-PD-1 and ZA exhibits a synergistic anti-HCC effect. The synergistic mechanism may be related to the polarization effect of ZA on macrophages. In addition, the impact of the change of ZA administration frequency on its anti-HCC effect is also worthy of attention.

## Data Availability

The datasets used and/or analyzed during the current study are available from the corresponding author on reasonable request.
